# Transporter-Guided Delivery of Nanoparticles to Improve Drug Permeation across Cellular Barriers and Drug Exposure to Selective Cell Types

**DOI:** 10.3389/fphar.2018.00027

**Published:** 2018-01-26

**Authors:** Longfa Kou, Yangzom D. Bhutia, Qing Yao, Zhonggui He, Jin Sun, Vadivel Ganapathy

**Affiliations:** ^1^Department of Cell Biology and Biochemistry, Texas Tech University Health Sciences Center, Lubbock, TX, United States; ^2^Municipal Key Laboratory of Biopharmaceutics, Department of Pharmaceutics, Wuya College of Innovation, Shenyang Pharmaceutical University, Shenyang, China

**Keywords:** plasma membrane transporters, nano-drug delivery systems, nanoparticles, targeted drug delivery, intestinal absorption, transfer across blood–brain barrier

## Abstract

Targeted nano-drug delivery systems conjugated with specific ligands to target selective cell-surface receptors or transporters could enhance the efficacy of drug delivery and therapy. Transporters are expressed differentially on the cell-surface of different cell types, and also specific transporters are expressed at higher than normal levels in selective cell types under pathological conditions. They also play a key role in intestinal absorption, delivery via non-oral routes (e.g., pulmonary route and nasal route), and transfer across biological barriers (e.g., blood–brain barrier and blood–retinal barrier. As such, the cell-surface transporters represent ideal targets for nano-drug delivery systems to facilitate drug delivery to selective cell types under normal or pathological conditions and also to avoid off-target adverse side effects of the drugs. There is increasing evidence in recent years supporting the utility of cell-surface transporters in the field of nano-drug delivery to increase oral bioavailability, to improve transfer across the blood–brain barrier, and to enhance delivery of therapeutics in a cell-type selective manner in disease states. Here we provide a comprehensive review of recent advancements in this interesting and important area. We also highlight certain key aspects that need to be taken into account for optimal development of transporter-assisted nano-drug delivery systems.

## Introduction

Plasma membrane transporters are critical for the nutrition of all mammalian cells; they provide glucose, amino acids, vitamins, purines and pyrimidines, ions, and other essential nutrients to the cells. In addition to their physiological substrates, they also interact with a variety of therapeutic drugs, in many cases as transportable substrates; as such, these transporters are critical determinants of drug efficacy and drug safety, including drug absorption, distribution, disposition, adverse drug reactions, drug–drug interactions, and therapeutic efficacy (Franke and Sparreboom, 2010; Zolk and Fromm, 2011; Bhutia et al., 2016). To date, more than 400 cell-surface transporters have been identified in human cells at the molecular level and functional level, which are classified into two major super families: ATP-binding cassette (ABC) transporters and solute carriers (SLC) (Giacomini et al., 2010). The transporters expressed in the intestine, liver, kidney, placenta, blood–brain barrier, and blood–retinal barrier have received special attention because of their critical role in the handling of nutrients as a component of cellular metabolism and also because of their involvement in the absorption and elimination of drugs. Many of these transporters feature prominently in drug development; it is mandatory that any new drug in development pipeline has to be evaluated for its interaction with a selective set of plasma membrane transporters as a prerequisite for approval by Food and Drug Administration. Plasma membrane transporters have also been shown to be useful in oral delivery of drugs in the form of prodrugs as evidenced from the successful exploitation of specific transporters to improve the oral bioavailability of the prodrugs such as valacyclovir (Ganapathy et al., 1998), valganciclovir (Sugawara et al., 2000), and gabapentin enacarbil (Stephen et al., 2004).

With the development of nanotechnology and nanomaterials, enormous progress on nano-drug delivery systems has been made. There is substantial and convincing evidence for enhanced efficacy of nanosized materials in oral drug delivery, cancer therapy, and site-specific drug delivery (Rieux et al., 2013; Yun et al., 2013; Dai et al., 2016; Date et al., 2016; Yao et al., 2017a,b). Some of these have already been approved for use in clinics (e.g., Abraxane, Doxil, Genexol-PM). Nano-drug delivery systems, collectively called nanoparticles, can encapsulate drugs, imaging agents, proteins, and genes. Nanoparticles can improve the solubility and the stability of encapsulated cargos, and even transfer across biological barriers such as the intestinal tract or blood–brain barrier. As for cancer therapy, chemotherapeutics-loaded nanoparticles can accumulate in tumors due to enhanced uptake and retention in tumor cells. However, conventional nanoparticles always exhibit off-target adverse effects; Even though nanoparticles improve the permeation efficacy across biological barriers, the improvement is not sufficiently large enough, hence requiring increased administration doses, which naturally increases the chances and the risk of unwanted side effects and toxicity.

**FIGURE 1:**
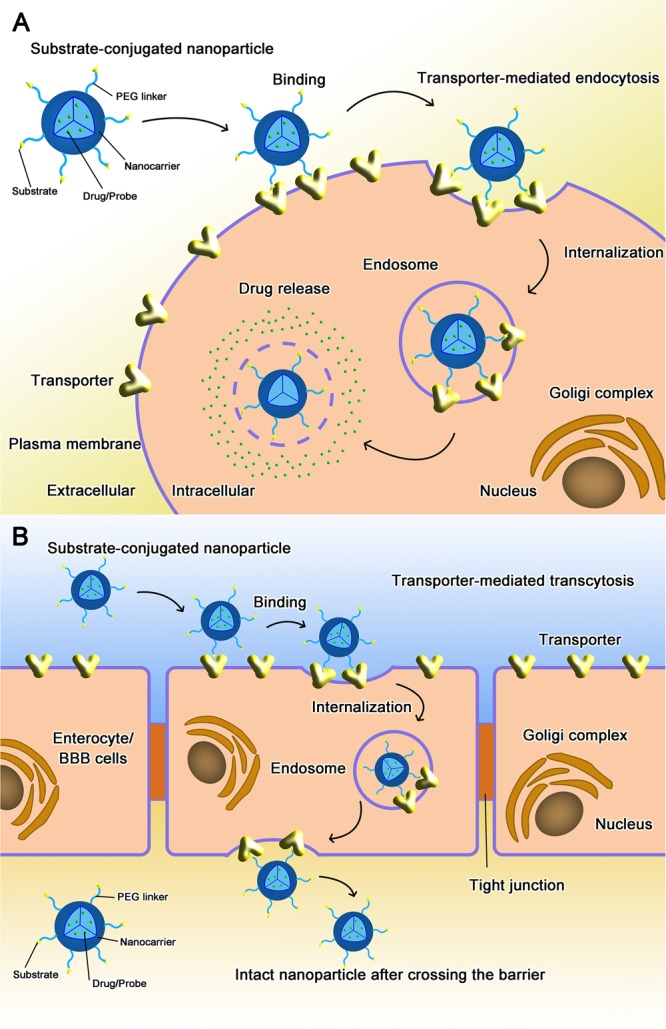
Transporter-assisted nanoparticles for **(A)** increased site-specific absorption and **(B)** enhanced permeation across a biological barrier.

Targeted nano-drug delivery systems conjugated with specific ligands could enhance the efficacy of drug delivery. Most transporters have a site-specific expression, which provide ideal targets for drug delivery to increase uptake at specific site (**Figure 1A**) or enhance permeation across biological barriers such as the blood–brain barrier (**Figure 1B**). In the intestinal tract, specific nutrient transporters are highly expressed to mediate the absorption of diet-derived nutrients; these transporters have been exploited for oral drug delivery to enhance bioavailability of therapeutics (**Table 1**). Tumor cells have an increased demand for nutrients such as glucose, amino acids and vitamins to support their malignant proliferation. As most of the nutrients are water-soluble, they can’t simply diffuse into cells across the lipid bilayer that constitutes the plasma membrane; they need selective cell-surface transporters to enter the cells. Tumor cells upregulate certain selective transporters in the plasma membrane to meet their increased demands for nutrients. Such transporters have been used as targets as delivery systems for anticancer therapeutics to effectively mediate tumor-cell selective drug delivery, thereby increasing the drug efficacy against the tumors (**Table 2**). Tumors residing behind biological barriers present an additional problem in effective treatment with drugs; the chemotherapeutics need to cross the biological barrier before they encounter the tumor cells. Brain tumors represent such an example. Drugs targeted for the treatment of brain tumors have to cross the blood–brain barrier before getting into the target cells. As the common nutrients necessary for the growth and survival of normal brain cells face the same issue, selective transporters are expressed at high levels in endothelial cells that constitute the blood–brain barrier. Examples of these transporters include GLUT1 (glucose transporter 1 or SLC2A1) for glucose transport, LAT1 (system L amino acid transporter 1; SLC7A5) for amino acid transport, and ChT1 (choline transporter 1; SLC5A7) for choline transport. These transporters have been utilized as an effective means to facilitate the transfer of therapeutic drugs across this biological barrier and hence for optimal therapy of tumors such as glioma that reside enclosed within the barrier (**Table 3**).

**Table 1:** Transporter-targeted nanoparticles for enhanced oral absorption.

Transporter	Gene	Substrate	Carrier	Drug	Reference
SMVT	SLC5A6	Biotin	Conjugates	Peptide	Ramanathan et al., 2001a,b
			Conjugates	Camptothecin	Minko et al., 2002
			Liposomes	Insulin	Zhang et al., 2014
			Solid lipid nanoparticles	Oridonin	Zhou et al., 2015
ASBT	SLC10A2	Deoxycholic acid	Conjugates	LMWH	Lee et al., 2001, 2006; Kim et al., 2007
		Deoxycholic acid	Conjugates	Insulin	Lee et al., 2005a
		TetraDOCA	Conjugates	LHT7	Alam et al., 2014
		Taurocholic acid	Micelles	Docetaxel	Khatun et al., 2013
OCTN2	SLC22A5	L-Carnitine	Nanoparticles	Paclitaxel	Kou et al., 2017c

**Table 2:** Transporter-targeted nanoparticles for increased site-specific absorption.

Transporter	Gene	Substrate	Carrier	Drug	Site	Reference
GLUT1	SLC2A1	2-Deoxy-D-glucose	DMSA-DG NPs	γ-Fe _2_O_3_	Tumor	Shan et al., 2012
		Glucose	Nanoparticles	Coumarin 6	Brain	Xie et al., 2012
GLUT4	SLC2A4	Glucose	Quantum dots		Muscle	Yeh et al., 2014
SMVT	SLC5A6	Biotin	Dendrimer	FITC	Tumor	Yang et al., 2009; Yellepeddi et al., 2009
			Dendrimer	Cisplatin	Ovarian cancer	Yellepeddi et al., 2011
			Pullulan acetate nanoparticles	Doxorubicin	Tumor	Na et al., 2003
			Polymer micelles	Doxorubicin	Tumor	Kim et al., 2012
			Erythrocytes	Methotrexate	Liver	Mishra and Jain, 2002
			Cubosomes	Paclitaxel, MO-Fluo	Tumor	Aleandri et al., 2015
			Polyurethane-urea nanoparticles	Sunitinib/phenoxodiol, plasmid DNA	Hepatocellular carcinoma	Morral-Ruíz et al., 2015
ATB^0,+^	SLC6A14	Lysine	Liposomes	Docetaxel	Liver cancer	Luo et al., 2016
		Aspartate	Liposomes	Docetaxel	Lung cancer	Luo et al., 2017
LAT1	SLC7A5	Glutamate	Nanoparticles	Paclitaxel	Breast cancer	Li et al., 2017
MCT1	SLC16A1	β-hydroxybutyrate	Solid lipid nanoparticles	Docetaxel	Brain	Venishetty et al., 2013
OCTN2	SLC22A5	L-Carnitine	Nanoparticles	5-Fluorouracil	Colon cancer	Kou et al., 2017b

**Table 3:** Transporter-targeted nanoparticles to enhance blood–brain barrier permeation and increase glioma targeting for optimal anti-glioma therapy.

Transporter	Gene	Substrate	Carrier	Drug	Reference
GLUT1	SLC2A1	2-Deoxy-D-glucose	Nanoparticles	Paclitaxel	Jiang et al., 2014b
		D-Glucosamine	Nanoparticles	Paclitaxel	Jiang et al., 2014a
		Dehydroascorbic acid	Micelles	Paclitaxel	Shao et al., 2014
ChT1	SLC5A7	Choline derivatives	Dendrimers	Plasmid DNA	Li et al., 2011
			Dendrimers	DTPA-Gd	Li et al., 2013b
			Micelles	Doxorubicin/Plasmid DNA	Li et al., 2013a
			Micelles	Doxorubicin	Li et al., 2015
LAT1	SLC7A5	Glutamate	Liposomes	Docetaxel	Li et al., 2016
OCTN2	SLC22A5	L-Carnitine	Nanoparticles	Paclitaxel	Kou et al., 2017a
SVCT2	SLC23A2	Vitamin C	Micelles	Rhodamine	Salmaso et al., 2009

Cell-surface receptors have also been used as targets for drug delivery. It should be noted, however, that there are at least a couple of differences between transporters and receptors when utilized as targets for nano-drug delivery systems: (i) The substrates of transporters are small molecules, which are stable, easily modifiable, and have no or little immunogenicity and steric hindrance whereas most of ligands for receptors are macromolecules (e.g., LDL, transferrin), and (ii) Transporters usually have broad substrate selectivity whereas the ligands for the receptors are much more specific. These differences could actually offer certain advantages in selecting cell-surface transporters for nano-drug delivery systems as it provides multiple choices in terms of ligands for modification of the surface of the nanoparticles to target the transporters (Kou et al., 2017b). Lack of immunogenicity of such ligands is also an added advantage.

## Effects of Physicochemical Properties of Nanoparticles on their Interaction with Transporters

### Effects from Conventional Properties of Nanoparticles

There is very little information in published literature with regard to the effects of nanoparticles’ physicochemical properties on their interaction with transporters, but their effects on the cellular uptake of nanoparticles have been investigated (Sahay et al., 2010; Kou et al., 2013; Howard et al., 2014). The smaller-sized nanoparticles are much more easily internalized into cells than the larger-sized nanoparticles. Positively charged nanoparticles prefer binding to negatively charged cell membrane, a process facilitated by electrostatic interaction. The shape of the nanoparticles is also a crucial factor in interaction with cells. In addition, the rate of uptake of nanoparticles is modulated by the angle of the particle binding to cell surface; elongated nanocarriers with a small binding angle generally show increased uptake.

In the case of intravenous administration, the half-life of nanoparticles in circulation is an important consideration. The particle size should not be too small; otherwise, the nanoparticles would undergo filtration at the renal glomerulus and consequently would get eliminated. The particle size should not be too large either; otherwise, the nanoparticles would promote thrombosis. The surface potential of the nanoparticles should be negative in order to increase their half-life in circulation.

### Effects from Modes of Ligand/Substrate Linkage to Nanoparticles

Poly (ethyleneglycol) (PEG) is often used as a linker to decorate the ligand/substrate on the surface of the nanoparticles; the linker distances the ligand/substrate from the surface and provides flexibility for more efficient interaction with the transporter. We manipulated the length of PEG for conjugation of L-carnitine to investigate the role of PEG linker in the interaction of the conjugated substrate with OCTN2; the uptake of L-carnitine-conjugated nanoparticles increased with the length of PEG to a certain degree, but then decreased with further increase in the length (Kou et al., 2017a). A similar phenomenon was also observed by other investigators (Xie et al., 2012; Li et al., 2017).

As conjugation of the ligand/substrate to the surface of the nanoparticles involves covalent bonding, the structure of the ligand/substrate after conjugation determines its interaction with the transporter. Thus, the selection of the functional groups in the ligand/substrate for covalent modification is critical. Kharya et al. (2013) developed phenylalanine-conjugated nanoparticles to target LAT1. However, as the α-carboxyl group of phenylalanine, a key requirement for recognition by LAT1, was used for the conjugation, this mode of linkage decreased the affinity of the conjugated substrate to LAT1. On the other hand, the mode of conjugation could also be used to our benefit. Free aspartate or glutamate is not a substrate for ATB^0,+^ (amino acid transporter B^0,+^; SLC6A14) because the transporter does not tolerate negative charge on the side chains of its amino acid substrates. But, if the β-carboxyl group in the side chain of aspartate or the γ-carboxyl group in the side chain of glutamate is used for covalent linkage with the surface of the nanoparticles, the negative charge is lost, thus leaving the remainder of the molecule as a recognizable substrate by the transporter (Luo et al., 2017). Alternatively, derivatives of physiological substrates with higher affinity than the parent substrate could be used to improve interaction of the nanoparticles with the transporters (Li et al., 2011; Al-Hilal et al., 2014c).

Ligand/substrate density is another important parameter that impacts on the interaction of the nanoparticles with the transporters. With appropriate ligand/substrate density, multivalent interaction between the nanoparticles and the transporters could be facilitated to optimize the uptake process. In our study, the cellular uptake of nanoparticles increased with the density of the conjugated L-carnitine increasing from 0 to 10% (Kou et al., 2017c). However, when the substrate density increased further to 20%, the uptake was decreased, suggesting that if the density of the substrate is too high, it might cause steric hindrance, thus interfering with the interaction of the nanoparticles with the transporter. Even though there was an optimal L-carnitine density for maximal interaction of the nanoparticles with OCTN2 in our study, there are cases where the uptake of nanoparticles kept on increasing with increasing density of the ligand within the range examined (Shao et al., 2014). This phenomenon obviously depends on the size of the ligand/substrate and whether or not the density of the ligand/substrate is examined in an appropriate range.

The chemical nature of the ligand/substrate, the length of the linker, and the density of the ligand/substrate collectively determine the affinity of the nanoparticles to the cell-surface target (Howard et al., 2014). In general, the higher the affinity of the nanoparticles to the cell-surface target, the greater is the cellular uptake of the nanoparticles; but there are exceptions to this general finding (Zern et al., 2013).

## Transporter-Assisted Nano-Drug Delivery Systems

### GLUT1/SLC2A1 (Glucose Transporter 1/Solute Carrier Family 2, Member A1)

Facilitative glucose transporters (GLUTs) are responsible for the transport of glucose from the circulation into target cells. As these are facilitative, they also function in the release of glucose from the cells into circulation in certain tissues (e.g., liver and small intestine). GLUT1, a representative member in GLUT family, is expressed ubiquitously; it is the transporter responsible for glucose uptake in erythrocytes and also for glucose transfer across the blood–brain barrier; it is also the transporter that supports glucose delivery into tumor cells as it is overexpressed in tumors (Wood and Trayhurn, 2003; FengMing et al., 2006). Glioma is one of the most malignant brain tumors in adults with high morbidity and mortality. It is a great challenge to effectively deliver therapeutics into glioma. The blood–brain barrier is the major hurdle for drug delivery into brain as it prevents the entry of >98% of small molecule drugs into brain (Pardridge, 2003; Agarwal et al., 2011; Allhenn et al., 2012). Even though the permeability of the barrier is compromised to some extent in glioma, the barrier still constitutes a significant obstacle for the delivery of drugs to treat glioma. The current treatment approach for glioma is surgical resection of accessible tumors; however, due to the infiltrative nature of the glioma, it is difficult to distinguish the tumor from the surrounding normal tissue for efficient resection of the tumor, which often leads incomplete resection and hence increases the chances for recurrence of the tumor. This necessitates the use of chemotherapy or adjunctive chemotherapy after surgery. GLUT1, highly expressed in both blood–brain barrier endothelial cells and in glioma cells, provides an ideal target for drug delivery to treat glioma.

Poly(ethylene glycol)-co-poly(trimethylene carbonate) nanoparticles functionalized with 2-deoxy-D-glucose (DGlu-NP) have been used successfully for glioma therapy by targeting the blood–brain barrier as well as the tumor (Jiang et al., 2014b). Compared to the non-modified nanoparticles, the uptake of DGlu-NP on RG-2 cells (a rat glioma cell line) was significantly increased, and could be inhibited by free glucose, suggesting that DGlu-NP was recognized by GLUT1 for enhanced cellular uptake. Furthermore, DGlu-NP accumulated to a greater extent in glioma than in surrounding normal tissue and also showed better anti-glioma efficiency *in vivo*. Similarly, D-glucosamine-modified nanoparticles also targeted GLUT1 and displayed high anti-glioma efficacy both *in vitro* and *in vivo* (Jiang et al., 2014a). To optimize this strategy, dehydroascorbic acid was used to functionalize nanodevices to target GLUT1 (Shao et al., 2014). Unlike the bidirectional transport of D-glucose by GLUT1, the transport of dehydroascorbic acid by GLUT1 is unidirectional because when this GLUT1 substrate gets into cells, it is reduced into ascorbic acid and gets trapped within the cells. This difference makes the drug delivery to glioma more efficient with dehydroascorbic acid-functionalized nanoparticles. The smart nanodevice used in this particular study was fabricated with a crosslinker containing disulfide bond, which stabilizes the nano-structure in circulation but gets destabilized inside the tumor cells to release the drugs due to the high cellular levels of glutathione.

GLUT1 is expressed at many-fold higher levels in almost all tumors to support the aerobic glycolysis (also called the Warburg effect), a hallmark of all cancers. This is the basis of the effective use of ^18^F-fluoro-2-deoxy-D-glucose in positron emission tomography in *in vivo* tumor diagnosis. Thus, this transporter can be used not only for tumor-specific drug delivery but also for tumor-specific delivery of imaging probes. Shan et al. prepared γ-Fe_2_O_3_ nanoparticles coated with dimercaptosuccinic acid and modified with 2-deoxy-D-glucose (γ-Fe_2_O_3_@DMSA-DG NPs) to target to GLUT1-overexpressed breast cancer for tumor imaging (Shan et al., 2012). It was found that the acquired MRI T2 signal intensity of breast cancer cells increased significantly when treated with γ-Fe_2_O_3_@DMSA-DG NPs, indicating that the nanoparticles could serve as a MRI agent for better tumor imaging. The validity of the same strategy was also confirmed with a cervical cancer cells (Xiong et al., 2012).

The size of the linkers used to link glucose onto the surface of the nanoparticles impact on the interaction of the ligand with GLUT1. Xie et al. (2012) prepared a series of glucose-modified liposomes using polyethylene glycols (PEG) with different chain lengths (200, 400, 1000, 2000) as the linkers. The qualitative and quantitative biodistribution assay in mice showed that the targeted liposomes using PEG1000 as the linker achieved highest brain accumulation.

GLUT4 is also a facilitative GLUTs, but unlike GLUT1, this transporter is expressed only in cardiac muscle, skeletal muscle and adipocytes; it is also the only facilitative GLUTss that responds to insulin (Wood and Trayhurn, 2003). Insulin acts through its cell-surface receptors in these tissues and the resultant intracellular signaling pathways promote translocation of GLUT4-containing vesicles from intracellular pools to the plasma membrane. Yeh et al. (2014) developed glucose-modified quantum dots (Glc-QDs) to facilitate the nanoparticle-based therapeutics and diagnostics. In the insulin-stimulated C2C12 muscle cells, the uptake of Glc-QDs was significantly increased compared to control cells without the insulin stimulation. The involvement of GLUT4 was confirmed by the ability of 2-deoxy-D-glucose to suppress the uptake of Glc-QDs.

### SMVT/SLC5A6 (Sodium-Dependent Multivitamin Transporter/Solute Carrier Family 5, Member A6)

Sodium-dependent multivitamin transporter (SMVT/SLC5A6) is an important transporter obligatory for the uptake of the vitamins biotin and pantothenate; it is highly expressed in placenta, intestine, brain, liver, lung, kidney and heart (Azhar et al., 2015). Biotin is a water-soluble vitamin, which is absorbed in the intestine via SMVT (Balamurugan et al., 2005). Biotinylated nanoparticles could therefore target to SMVT for improved oral absorption of drugs. Ramanathan et al. (2001a) linked biotin to a nonapeptide (R.I.-K-Tat9) for improved uptake of the peptide in the intestinal cell line Caco-2. The uptake increased threefold with biotin compared to without biotin. The addition of PEG (polyethylene glycol) to R.I.-K(biotin)-Tat9 increased the uptake efficacy even further (Ramanathan et al., 2001b). The participation of SMVT in the uptake process was supported by the findings that SMVT substrates inhibited the uptake to a significant extent. Minko et al. (2002) conjugated PEG to camptothecin, and then linked biotin to the PEG side of the conjugate. The biotinylated conjugate also could increase the transfer across the Caco-2 monolayer involving SMVT due to the presence of biotin in the conjugate. Zhang et al. (2014) prepared biotinylated liposomes as potential carrier for the oral delivery of insulin; a significant hypoglycemic effect after oral administration of the biotinylated liposomes was observed with a bioavailability fivefold greater than with unmodified liposomes. Zhou et al. (2015) constructed biotinylated nanostructured lipid carriers for oral delivery of oridonin, a natural compound with anti-inflammation and anti-cancer activities. Both the biotinylated and conventional nanoparticles increased the oral bioavailability of oridonin, but the effect was greater with biotinylated naoprticles than with unmodified nanoparticles.

SMVT expressed in non-intestinal tissues has also been targeted for site-specific drug delivery. Mishra and Jain (2002) developed biotinylated methotrexate-loaded erythrocytes for liver-targeted delivery. The uptake *in vitro* was enhanced more than twofold compared to unmodified erythrocytes. *In vivo* studies showed that the therapeutic index of liver targeting was enhanced threefold with biotinylation. As many other vitamin transporters, SMVT is also expressed at higher levels in tumors than in normal tissues. Yang et al. (2009) and Yellepeddi et al. (2009) prepared biotin-conjugated and FITC-labeled PAMAM [poly(amido)amine dendrimers]. The uptake of biotin-conjugated PAMAM into SMVT-positive cancer cells was significantly increased compared to that of unmodified PAMAM, and free biotin inhibited the uptake of biotin-conjugated PAMAM. The same was true with cisplatin-loaded PAMAM (Yellepeddi et al., 2011). Na et al. (2003) prepared biotin-conjugated pullulan acetate nanoparticles with varying density of biotin on the surface of the nanoparticles for anti-cancer drug delivery. The uptake of biotin-conjugated nanoparticles was significantly increased in HepG2 cells, and the uptake enhancing effect was greater with increasing density of biotin on the nanoparticle surface. Kim et al. (2012) prepared biotin-conjugated polymeric micelles for active and pH-sensitive tumor targeting. The biotinylated micelles showed higher uptake than unmodified micelles in tumor cells.

The overexpression of SMVT in cancer cells is also of use for simultaneous delivery of not only anticancer drugs but also imaging probes. Aleandri et al. (2015) prepared biotinylated cubosomes, which were used to simultaneously transport anticancer drugs and fluorescent dye into cancer cells. This strategy has applications cancer treatment aiding in detection, therapy, and monitoring of the therapeutic response. Morral-Ruíz et al. (2015) used polyurethane-urea nanoparticles for targeted anticancer drugs and plasmid DNA delivery to hepatocellular carcinoma and showed that biotinylation of the nanoparticles improved cargo delivery and also reduced off-target drug exposure.

### ChT/SLC5A7 (Choline Transporter/Solute Carrier Family 5, Member A7)

Choline is an essential compound involved in the synthesis of the neurotransmitter acetylcholine and the membrane phospholipid phosphatidylcholine. Choline transporters (ChT) are responsible for cellular uptake of this compound. There are two distinct classes of ChTs, primarily based on Na^+^-dependence and the affinity for choline (Allen and Lockman, 2003). The Na^+^-dependent low-affinity transporter is expressed ubiquitously in the body whereas the Na^+^-dependent high-affinity transporter is expressed in pre-synaptic cholinergic nerve terminals. The Na^+^-independent transporter is responsible for choline transfer across the blood–brain barrier.

The structural components in choline necessary for interacting with the transporter include the positively charged quaternary ammonium group and the hydroxyl group (Geldenhuys et al., 2005); therefore, neither of these functional groups is useful for linking to the surface of nanoparticles as a means to target the choline transporters to improve the cellular uptake of nanoparticles. This hurdle was overcome with the synthesis of appropriate derivatives of choline that retained the ability to interact with ChTs (Li et al., 2011). The derivative with the highest affinity to ChT was linked to dendrimers to improve the delivery of plasmid DNA as a cargo (DGL-PEG-CD) across the blood–brain barrier (Li et al., 2011).

Gliomas are the most common type of primary central nervous system cancer in adults (Huse and Holland, 2010). The glioma cells have an increased demand for choline to synthesize membrane phospholipids essential for cell proliferation. Surgical resection is the most common treatment, and a precise delineation of tumor margins is necessary for the success of this surgical therapy. Li et al. (2013b) utilized the same DGL-PEG-CD system linked with DTPA-Gd (Gd-diethyltriaminepentaacetic acid), a contrast agent, for precise detection of gliomas. Based on the ChT targeting, the nanoprobe was able to cross the blood–brain barrier and accumulate in glioma cells. The selective accumulation of the contrast agent in gliomas enables clear identification of the margins of the tumor distinguishing it from the surrounding normal tissue with magnetic resonance imaging (MRI).

It has been shown that a combination of chemotherapy and gene therapy could be synergistically effective for cancer therapy (Conde et al., 2016; Hu et al., 2017; Tuppurainen et al., 2017; Yang et al., 2017). To deliver plasmids carrying therapeutic genes into tumors, a choline derivative-modified co-delivery system has been evaluated which targets ChT; the system was used to deliver a plasmid encoding TRAIL (human tumor necrosis factor-related apoptosis-inducing ligand) and doxorubicin (Li et al., 2013a). The efficacy of this co-delivery system was examined in the glioma cell line U87 MG *in vitro* and then in mouse xenografts *in vivo*. Choline derivative-modified micelles were also effective in improving glioma therapy via ChT dual-targeted strategy (Li et al., 2015).

### ATB^0,+^/SLC6A14 (Amino Acid Transporter B^0,+^/Solute Carrier Family 6, Member A14)

Tumor cells exhibit metabolic reprogramming and show increased requirement for all amino acids, particularly to glutamine (“glutamine addiction”). The upregulation of several amino acid transporters in tumors has been documented; one of these transporters is ATB^0,+^ (SLC6A14), which exhibits functional features that are uniquely suited to satisfy the increased demands for amino acids in tumor cells (Ganapathy and Ganapathy, 2005; Bhutia et al., 2014). This transporter has a broad substrate selectivity and is highly concentrative. Though it is expressed only at levels in normal tissues, its expression is markedly elevated in many cancers, particularly in colon cancer (Gupta et al., 2005), cervical cancer (Gupta et al., 2006), estrogen receptor-positive breast cancer (Fujisaki et al., 2008), and pancreatic cancer (Bhutia et al., 2014). The upregulation is not universal in all cancers, but the tumors that are positive for increased expression of this transporter could be treated with pharmacological blockade of this transporter as a means to prevent entry of amino acids into tumor cells and thereby essentially starving the tumor cells to death.

Using ATB^0,+^ as the target, we developed amino acid-conjugated liposomes for enhanced anticancer efficacy and decreased off-target side effects (Luo et al., 2016, 2017). Three different amino acids, glycine, aspartate, and lysine, were evaluated individually to determine their relative efficiency in targeting ATB^0,+^. Lysine-conjugated liposomes showed the best efficacy. The uptake of lysine-conjugated liposomes was enhanced in HepG2 cells (a human hepatocellular carcinoma cell line), which show abundant expression of ATB^0,+^; the uptake was inhibited by free glycine and lysine (Luo et al., 2016). However, when L929 cells (a mouse fibroblast cell line) which have low levels of ATB^0,+^ expression, lysine-conjugated liposomes failed to show any improvement in uptake over naïve liposomes without lysine. In HepG2 cells, the uptake of lysine-conjugated liposomes decreased when the experiments were conducted at 4°C, one of the features that provides evidence for the involvement of transporter-mediated process in the uptake. Experiments with endocytosis inhibitors revealed that ATB^0,+^-assisted uptake of lysine-conjugated liposomes involved endocytosis. *In vivo* studies with HepG2 xenografts in mice documented selective accumulation of lysine-conjugated liposomes compared to unmodified liposomes in tumors. We also performed detailed analysis of the involvement of ATB^0,+^ in the uptake of lysine-conjugated liposomes. The transporter is obligatorily dependent on Na^+^ and Cl^-^ for its function. We were able to demonstrate that the uptake of lysine-conjugated liposomes was decreased in the absence of not only Na^+^ but also Cl^-^; this dependence on Na^+^ or Cl^-^ was not evident in the case of unmodified liposomes. With molecular dynamic simulation, we also found that the binding energy of lysine-conjugated liposomes to the transporter decreased in the presence of Na^+^ and Cl^-^; this demonstrated that the presence of Na^+^ and Cl^-^ thermodynamically favored the interaction of lysine-conjugated liposomes with the transporter. Conjugation of aspartate instead of lysine also displayed similar characteristics (Luo et al., 2017). Free aspartate is not a substrate for this transporter because of the negative charge of the β-carboxyl group on the side chain, but when it is conjugated to liposomes via its β-carboxyl group, the ligand loses the negative charge on the side chain and is recognized as a substrate by ATB^0,+^.

### LAT1/SLC7A5 (System L Amino Acid Transporter 1/Solute Carrier Family 7, Member A5)

The amino acid transporter LAT1 (SLC7A5) mediates cellular uptake of almost all neutral amino acids, but unlike ATB^0,+^, LAT1-mediated uptake is Na^+^-independent (Kanai et al., 1998). LAT1 prefers bulky neutral amino acids, which include branched chain amino acids and also aromatic amino acids. Its expression is also increased in multiple cancers, including breast cancer, cervical cancer, lung cancer, and prostate cancer (Yanagida et al., 2001). Therefore, this transporter also has potential for use in targeted delivery of cancer therapeutics in the form of amino acid-conjugated nanoparticles. Several studies have documented the utility of this approach. Kharya et al. (2013) prepared phenylalanine-coupled solid lipid nanoparticles for glioma therapy with the goal of targeting LAT1 which is expressed in blood–brain barrier endothelial cells and also on glioma cells. Results of these studies showed increased delivery of anticancer drugs into glioma cells when the nanoparticles were modified to have phenylalanine on their surface. However, it was not clear whether the improved uptake into tumor cells actually involved LAT1. The presence of free amino group and free carboxyl group on the α-carbon atom of the amino acid is critical for recognition of the substrate by LAT1 (Rautio et al., 2013). In the study by Kharya et al. (2013), the α-carboxyl group of phenylalanine was used to link nanoparticles, a maneuver likely to interfere with the interaction of the modified ligand with LAT1. Despite this obvious hurdle, phenylalanine-conjugated nanoparticles showed improved uptake in glioma cells; it is possible that the hydrophobicity of phenylalanine on the surface of the nanoparticles contributed somehow to the improved uptake. To avoid the loss of affinity of modified amino acid to LAT1, we linked the γ-carboxyl of glutamate to the surface of liposomes to make LAT1-targeted liposomes as this method of linking maintained the α-amino group and the α-carboxyl group intact for LAT1 recognition (Li et al., 2016). Compared to the unmodified liposomes, glutamate-conjugated liposomes showed significant increase in cellular uptake and cytotoxicity in C6 glioma cells. *In vivo* studies showed that LAT1-targeted liposomes were able to cross the blood–brain barrier very effectively. These data suggested that glutamate conjugation to nanodevices via its side chain preserves the interaction of the modified amino acid with LAT1. We evaluated the use of such liposomes for tumor-targeted drug delivery (Li et al., 2017). Glutamate-conjugation significantly increased the cellular uptake and cytotoxicity of nanoparticles in HeLa (a human cervical cancer cell line) and MCF7 (a human estrogen receptor-positive breast cancer cell line) cells, both of which show robust expression of LAT1. *In vivo*, glutamate-conjugated nanoparticles showed enhanced accumulation of drugs in tumors and also increased antitumor efficacy compared to the unmodified nanoparticles.

### ASBT/SLC10A2 (Apical Sodium-Bile Salt Transporter/Solute Carrier Family 10, Member A2)

Bile acids are obligatory for digestion and absorption of dietary fat and fat-soluble vitamins; these are synthesized in the liver from cholesterol and secreted into bile to aid fat digestion in the intestinal tract (Hofmann and Hagey, 2008). These bile acids undergo enterhepatic circulation, a phenomenon in which the same bile acid pool is recirculated multiple times between the intestine and liver. After helping the digestion of dietary fat in the small intestine, a majority of bile acids are absorbed back in the ileum to enter into portal circulation form where they are taken into hepatocytes for secretion into bile again. This process involves several transporters, one in the apical membrane of ileal enterocytes, one in the basolateral membrane of ileal enterocytes, one in the sinusoidal membrane of hepatocytes, and one in the canalicular membrane of hepatocytes (Dixon and Williamson, 2011). ASBT (apical sodium-dependent bile salt transporter, SLC10A2) expressed on the apical membrane of the enterocytes in ileum has received significant attention as a potential target for drug absorption (Dawson et al., 2009). This is primarily due to the fact that ASBT is an efficient and high-capacity transporter as is evident from its physiological role in successfully accomplishing the intestinal absorption of ∼25 g of bile acids per day with only <5% loss in the feces (Dawson et al., 2009). A lot of information is available on the transport mechanism of this transporter. At the initial step in transporting substrates, ASBT exists in an outward-facing conformation with a cavity formed by the transmembrane domains; this cavity constitutes the active site for the binding of the bile acid substrate and the co-transported ion Na^+^, following which the conformation shifts to an inward-facing configuration to facilitate the release of the substrate and the co-transported ion inside the cell (Hu et al., 2011). As bile acids are highly hydrophobic, they cannot exist in solution in free form; they bind to a protein inside the enterocytes of the ileum and then get exported out across the basolateral membrane into the portal circulation via OST-α/β (Gong et al., 1994; Rao et al., 2008; Lm et al., 2013). ASBT has been evaluated in several studies for its potential as a drug delivery system, not only for prodrugs by coupling therapeutic drugs to bile acids but also for nanoparticles by conjugating bile acids onto the surface of these nanoparticles.

A considerable amount of work has been done on the use of bile acid-conjugated macromolecules for improved oral absorption (Lee et al., 2001, 2006; Kim et al., 2007). The first example was to improve oral bioavailability of heparin via ASBT; for this, the bile acid deoxycholic acid was conjugated to low molecular weight heparin to facilitate recognition of the modified heparin by ASBT (Lee et al., 2001). *In vivo* using non-human primates, it was found that bile acid-conjugated heparin was absorbed in the intestine at almost 10-fold higher levels than heparin alone (Kim et al., 2007). As the bile acid conjugate was hydrophobic, it had to be dissolved in an organic solvent to improve oral absorption; without the organic solvent, the improvement of bile acid-conjugated hepartin absorption was only twofold (Lee et al., 2006). The same bile acid-conjugated heparin was examined for transcellular transfer across the Caco-2 cell monolayer; again, the presence of the bile acid improved the transfer of heparin from the apical compartment to the basolateral compartment, and the process was inhibited by free bile acid, implicating ASBT involvement (Kim et al., 2007). The same strategy was also used for insulin to improve its oral bioavailability (Lee et al., 2005a).

The substrate-binding pocket in ASBT is much bigger than bile acids, suggesting that it could interact and accommodate larger substrates with higher affinity and efficiency than the bile acids themselves (Park et al., 2010; Al-Hilal et al., 2014c). Subsequent studies showed that it was indeed the case. When several oligomers of deoxycholic acid were examined, the tetrameric form of this bile acid showed the highest affinity for ASBT (Al-Hilal et al., 2014c). Conjugation of the tetrameric form of the bile acid to heparin increased the binding of the modified heparin to the apical membrane of ileal enterocyte and its entry into cells via (Al-Hilal et al., 2014b). *In vivo*, oral administration of heparin conjugated to bile acid tetramer was able to abolish the majority of the coagulation-dependent tropism of fibrinogen and remarkably decrease hemorrhage (Al-Hilal et al., 2014a). Addition of taurocholic acid, another bile acid, to heparin along with the deoxycholic acid tetramer provided another advantage in heparin delivery; it improved the biological half-life (Alam et al., 2014). When combined with deoxycholylethylamine, a potent absorption enhancer (Lee et al., 2005b), the same strategy could not only increase the oral bioavailability but also prolong the biological half-life (Alam et al., 2014).

ASBT is a useful target not only for the delivery of drugs in the form of bile acid-conjugated nanoparticle but also for suppressing enterohepatic circulation of bile acids as a strategy to reduce digestion and absorption of dietary fat. This could be a means to prevent obesity and hyperlipidemia. Park et al. (2015) has succeeded in designing a novel hydrophilic ASBT inhibitor to prevent the ileal absorption of bile acids *in vivo*, thereby interrupting the enterohepatic circulation of bile acids. This inhibitor consisted of polyacrylic acid conjugated to tetrameric form of deoxycholic acid; this conjugate could effectively bind to ASBT in MDCK cells overexpressing the transporter; *in vivo*, this conjugate was able to inhibit high-fat diet-induced hyperlipidemia. The hydrophobic nature of deoxycholic acid favors preferential localization of this bile acid in the hydrophobic core of nanomicelles instead of localization on the outer surface, thus reducing the efficiency with which the bile acid-conjugated nanoparticles interact with ASBT. Taurocholic acid is more water soluble than deoxycholic acid; therefore, replacement of deoxycholic acid with taurocholic acid solves this problem (Khatun et al., 2013).

### MCT1/SLC16A1 (Monocarboxylate Transporter 1/Solute Carrier Family 16, Member A1)

MCT1 is a proton-coupled transporter for monocarboxylates, which include lactate as well as the ketone body β-hydroxybutyrate; this transporter is expressed in endothelial cells in the blood–brain barrier (Venishetty et al., 2013). Venishetty et al. (2013) explored β-hydroxybutyrate-grafted docetaxel-loaded solid lipid nanoparticles to enhance the drug distribution to brain. The uptake and cytotoxicity of β-hydroxybutyrate-grafted nanoparticles was significantly increased in brain endothelial cells compared to unmodified nanoparticles. Free β-hydroxybutyrate inhibited the uptake of β-hydroxybutyrate-grafted nanoparticles, a MCT1-targeted mechanism. β-Hydroxybutyrate-grafted nanoparticles could effectively increase docetaxel distribution into brain.

### OCTN2/SLC22A5 (Novel Organic Cation Transporter 2/Solute Carrier Family 22, Member A5)

L-carnitine (β-hydroxy-γ-trimethylaminobutyrate) is a highly polar zwitterionic molecule obligatory for the transfer of long-chain fatty acids across the inner mitochondrial membrane for subsequent β-oxidation. L-Carnitine can be synthesized from lysine endogenously, but a majority of L-carnitine in the body is from intestinal absorption of dietary L-carnitine (Rebouche and Seim, 1998). OCTN2 is responsible for the transport of L-carnitine across plasma membrane in mammalian cells, and it expressed throughout the intestinal tract (Tamai, 2013). OCTN2 is a Na^+^-dependent, high-affinity transporter for L-carnitine with an apparent K_m_ about 10 μM (Pochini et al., 2013). L-Carnitine contains a hydroxyl group which can be used for chemical modification. We synthesized a series of prodrugs linking L-carnitine with gemcitabine for increased oral absorption (Wang et al., 2017). The oral bioavailability of one of the prodrugs could be increased up to 4.9-fold compared to gemcitabine. These results confirmed that OCTN2 could be used as a target for oral drug delivery system.

We then examined the utility of OCTN2 for nanoparticle delivery. We linked L-carnitine to the surface of the nanoparticles to prepare OCTN2-targeted nanoparticles (Kou et al., 2017c). L-Carnitine-conjugated nanoparticles (LC-PLGA NPs) targeting to OCTN2 enhanced oral bioavailability. There was an optimal value for the surface density of L-carnitine; we found 10% L-carnitine to provide the maxima enhancement of nanoparticle uptake. This phenomenon was also observed in other studies (Gu et al., 2008; Fakhari et al., 2011; Elias et al., 2013; Chu et al., 2016). The uptake of L-carnitine-conjugated nanoparticles in Caco-2 cells was inhibited by free L-carnitine and was also Na^+^-dependent. The uptake process involved endocytosis. The Na^+^-dependent manner of L-carnitine-conjugated nanoparticles was investigated using molecular dynamic simulation. The results showed that the binding energy of L-carnitine-conjugated nanoparticles to OCTN2 decreased in the presence of Na^+^. We proposed that the L-carnitine-conjugated nanoparticles and Na^+^ bind to OCTN2 to form a complex, which then induces membrane invagination and trigger the endocytosis process.

OCTN2 is highly expressed in brain capillary endothelial cells that constitute the blood–brain barrier (Kido et al., 2001; Inano et al., 2003; Berezowski et al., 2004; Miecz et al., 2008). We also found robust expression of this transporter in glioblastoma multiforme T98G cells (Kou et al., 2017a). Therefore, OCTN2 offers a potential target to improve the delivery of chemotherapeutics to brain. We tested the potency of L-carnitine-conjugated nanoparticles for glioma targeted drug delivery (Kou et al., 2017a). Conjugation of L-carnitine significantly increased the uptake of nanoparticles in the blood–brain barrier endothelial cell line hCMEC/D3 and glioma cell line T98G. Paclitaxel-loaded nanoparticles with conjugated L-carnitine showed improved anti-glioma efficacy. *In vivo* studies provided evidence for improved accumulation in brain following L-carnitine conjugation.

### SVCT2/SLC23A2 (Sodium-Coupled Vitamin C Transporter 2/Solute Carrier Family 23, Member A2)

Vitamin C (ascorbic acid) is a water-soluble vitamin, and transporters for vitamin C belong to SLC23 family (Bürzle et al., 2013). SVCT1 (SLC23A1) is expressed primarily in epithelial tissues such as small intestine, where it contributes to the absorption of the vitamin from dietary sources. The expression of SVCT2 is relatively broad, and it is found in blood–brain endothelial cells. Several studies have examined the potential use of SVCT2 in drug absorption, but little attention has been given to the potential use of this transporter in the delivery of nanoparticles. Salmaso et al. (2009) prepared vitamin C-conjugated micelles to target SVCT2 for brain drug delivery. *In vitro* results showed that vitamin C-conjugated micelles could increase cellular uptake and cytotoxicity against cancer cells compared to unmodified micelles. In the presence of free vitamin C, the uptake of such nanoparticles decreased, indicating SVCT2-targeted property.

### P-gp/ABCB1 (P-Glycoprotein/ATP-Binding Cassette Transporter Family, Member B1)

ATP-binding cassette (ABC) transporters is a major superfamily of membrane transporters, which bind and hydrolyze ATP to drive the efflux of various compounds out of cells. Several of the drugs that are used routinely in clinical practice are substrates for this transporter. This includes paclitaxel, docetaxel, and doxorubicin. The most-investigated member of the ABC transporter family is *P*-glycoprotein (P-gp/ABCB1/MDR1). As the primary function of MDR1 is to efflux drugs out of the cells, its utility in facilitating drug entry into cells remains doubtful. Nonetheless, there was a report that linking the substrate of *P*-gp to the surface of nanoparticles significantly increased the uptake of nanoparticles (Dreaden et al., 2014). The substrates examined are azithromycin, clarithromycin, and tricyclic ketolide. Furthermore, the uptake of azithromycin- and clarithromycin-conjugated nanoparticles was increased in the presence of *P*-gp inhibitors (cyclosporine A and verapamil) in *P*-gp overexpressed cells.

## Cellular Trafficking of Transporter-Targeted Nanoparticles

### The Intracellular Fate of Transporter-Targeted Nanoparticles

With physiological substrates, cell-surface transporters function simply as carriers, and the transport mechanism involves four steps: binding of the substrate on the outer surface of the membrane, conformational change in the transporter protein because of the substrate binding, transition of the substrate-bound transporter from the outward-facing configuration to an inward-facing configuration, and finally release the bound substrate inside the cells. In the absence of involvement of transmembrane ion gradients, the transport process is normally bidirectional. Involvement of ion gradients could favor the transport in one direction depending on the magnitude and direction of the ion gradient across the plasma membrane. Some transporters depend on ion gradients solely based on the ionic nature of a given substrate. OCTN2 is Na^+^-coupled if the zwitterionic L-carnitine is the substrate, but is Na^+^-independent if cationic molecules are substrates; in this case, L-carnitine transport is mostly unidirectional preferring movement of the substrate from outside to inside whereas organic cation transport could be in either direction (Tamai et al., 1998). Other factors are also involved in determining the directionality of the transport process. Transport of glucose via GLUT1 is bidirectional whereas transport of dehydroascorbic acid by the same transporter is almost unidirectional dictated by the immediate conversion of the transported substrate inside the cells into a non-substrate form (Shao et al., 2014).

**FIGURE 2:**
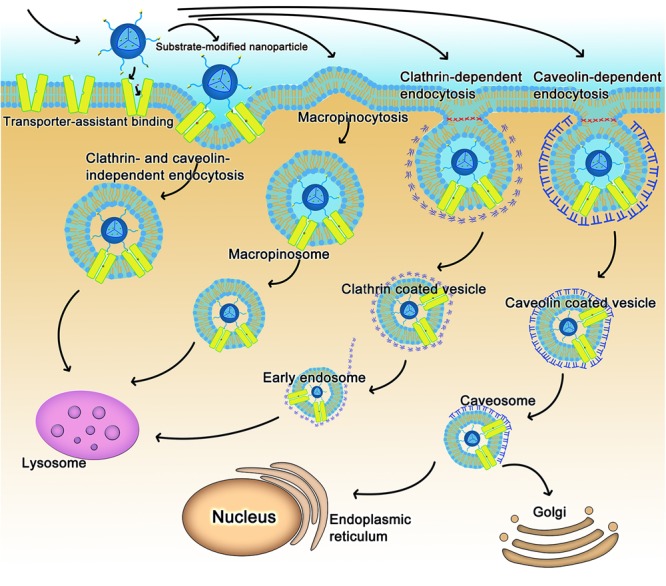
The intracellular fate of transporter-assisted nanoparticles based on transporter-mediated endocytosis.

As for nanoparticles or macromolecular conjugates, the transporter-assisted transfer process works differently; the mechanism is more like receptor-mediated transport. We have elucidated the probable mechanism of transporter-assisted uptake of nanoparticles (Luo et al., 2016; Kou et al., 2017c; Li et al., 2017). Substrate-conjugated nanoparticles bind to the targeted transporters on the outside along with co-transported ions if involved, but the macromolecular nature of the nanoparticles prevents the four-step transfer mechanism described above for small molecule substrates. The transporter stays trapped in the occluded conformation as a complex bound to the nanoparticles and co-transported ion, if any, which alters surface properties of the plasma membrane triggering membrane invagination along with the transporter complex. This initiates the endocytic process. Endocytosis can be classified as phagocytosis or pinocytosis based on the cell type and size of content (Sahay et al., 2010; Kou et al., 2013). In mammalian cells, phagocytosis occurs only in phagocytes, whereas pinocytosis occurs in all cells (Rappoport, 2008; Wang et al., 2011). Based on the proteins involved, endocytosis is further classified as clathrin-dependent endocytosis, caveolin-dependent endocytosis, macropinocytosis, and clathrin- and caveolin-independent endocytosis (Sahay et al., 2010; Kou et al., 2013). The endocytosis pathway could decide the fate of nanoparticles in transporter-assisted uptake. With the exception of caveolin-dependent endocytosis, other endocytic pathways involve fusion of endocytosed nanoparticles with lysosomes with subsequent release of the encapsulated drugs (**Figure 2**). If the cargo consists of proteins, peptides or plasmid DNA, this pathway might lead to inactivation of the cargo because of the involvement of lysosomal degrading enzymes. The caveolin-dependent endocytosis avoids the lysosomal compartment and hence the cargo remains intact and unaffected. This pathway is used by some pathogens such as viruses and bacteria (Pelkmans et al., 2002; Khalil et al., 2006). Free vitamin B_12_ enters cells via clathrin-dependent endocytosis; as such, vitamin B_12_-modified nanoparticles employ caveolin-dependent pathway (Fowler et al., 2013). These findings would be of use while designing nano-drug delivery systems for cargos such as DNA or proteins/peptides.

The nature of the transporter substrate used for conjugation in the design of nano-delivery systems might also determine the type of endocytic process. For example, dehydroascorbic acid as a GLUT1 substrate has no impact on the type of endocytic process that participates in the uptake of nanoparticles; caveolin-dependent endocytosis mediates the cellular uptake of nanoparticles irrespective of whether or not dehydroascorbic acid is conjugated on the surface of the nanoparticles (Shao et al., 2014). In contrast, conjugation of biotin to liposomes changes the endocytosis pathway (Zhang et al., 2014).

### The Fate of Transporters Targeted by Nanoparticles

**FIGURE 3:**
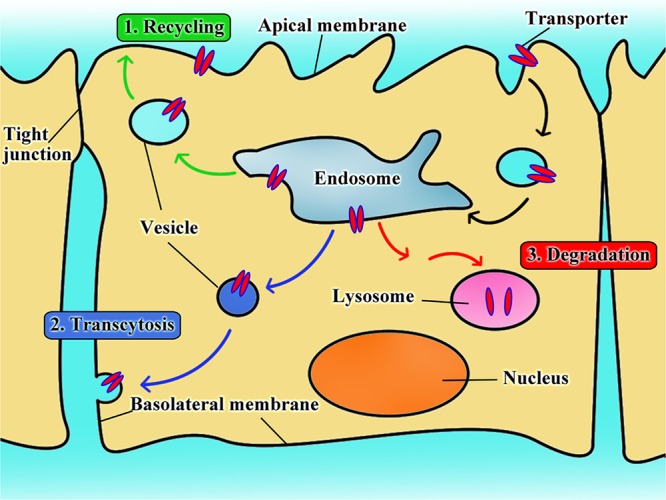
The intracellular fate of transporter when used as target for nanoparticles.

There is only limited information available on the fate of transporters in transporter-assisted uptake of nanoparticles. In analogy with the fates of cell-surface receptors in receptor-mediated endocytosis, there are potentially three different fates for the transporters in transporter-assisted uptake of nanoparticles (**Figure 3**): (i) the transporters follow the recycling route in which once the substrate-conjugated nanoparticles along with their cargo are dissociated from the transporter, the transporter recycles back to the plasma membrane; (ii) the transporters follow the transcytosis route in which they stay bound to the nanoparticles in the pinched-off vesicles that originate on one side of the cell, travel across the cell, and then fuse with the membrane on the other side of the cell to exit; (iii) the transporters follow the fate of the nanoparticles and their cargo where the transporters get degraded in endosome/lysosomes. Our studies show that all three routes play a role in different proportions depending not only on the transporter but also on the cell type. In one of our studies with LAT1-targeted nanoparticles with cultured cell lines, the amount of LAT1 protein was decreased at the plasma membrane but increased in cytoplasm at the beginning of the uptake process (Li et al., 2017). However, once the nanoparticles were removed from the uptake medium, the transporter density in the plasma membrane increased with concomitant decrease in the cytoplasm, thus providing evidence for the recycling route. In another study with OCTN2-targeted nanoparticles, oral administration of L-carnitine-conjugated nanoparticles in live animals led to the appearance of a significant amount of intact L-carnitine-conjugated nanoparticles appeared in the lymphatic system, providing evidence for the transcytosis route (Kou et al., 2017c). A similar phenomenon also occurred when nanoparticles were designed for enhancement of the transfer across the blood–brain barrier endothelial cells to target gliomas (Shao et al., 2014; Li et al., 2015; Kou et al., 2017a). In some of our studies, we also found evidence for the third route, namely degradation of the transporter inside the cells following uptake (Luo et al., 2016; Kou et al., 2017c).

## Summary and Outlook

There is overwhelming evidence in recent years for advantages of targeted nanoparticles in multiple fronts. Here we have reviewed the current literature on transporter-targeted nanoparticles. This is a relatively a new area in the drug delivery field. A critical analysis of the literature reveals definitive potential for transporter-targeted nanoparticles to deliver drug cargos in cell-type specific manner, to promote drug transfer across biological barriers, and to increase oral bioavailability of drugs.

The sheer number of transporters and, in many cases, their relatively broad substrate selectivity offer multiple advantages in the design of nanoparticles to target a specific transporter. First, there are choices for ligand selection, which could be important in terms of efficacy and feasibility. Second, there are opportunities to select the most desirable transporter for targeting depending on the purpose of drug delivery. If enhancement of oral delivery is desired, intestine-specific transporters could be selected for targeting. If delivery to brain is the goal, transporters in the blood–brain endothelial cells could be selected. The same is true for drug delivery to tumor cells. Third, there are also possibilities to select a ligand that could target more than one transporter as a means to increase the delivery efficacy. Amino acid transporters with overlapping substrate selectivity are ideal targets in this regard.

It has, however, to be borne in mind that the field of transporter-assisted nanoparticles is still at its infancy. There are examples of transporter-mediated prodrugs and conventional nanoparticles that have been approved by the US Food and Drug Administration (FDA), but none of the targeted nanoparticles, either cell-surface receptor-assisted or cell-surface transporter-assisted, has actually reached the market. This does not necessarily mean that the idea of transporter-assisted nanoparticles has not potential. The field itself is at its beginning stage and additional research is needed to validate the eventual success or failure of this approach.

## Author Contributions

VG and JS conceived the review, decided on the general outline, and finalized the manuscript. LK wrote the first draft of the manuscript. YB, QY, and ZH critiqued and revised the manuscript. All authors read the final version of the manuscript and approved it for submission.

## Conflict of Interest Statement

The authors declare that the research was conducted in the absence of any commercial or financial relationships that could be construed as a potential conflict of interest.
